# Increasing the level of cytoskeletal protein Flightless I reduces adhesion formation in a murine digital flexor tendon model

**DOI:** 10.1186/s13018-020-01889-y

**Published:** 2020-08-27

**Authors:** Jessica E. Jackson, Zlatko Kopecki, Peter J. Anderson, Allison J. Cowin

**Affiliations:** 1 Regenerative Medicine, Future Industries Institute, University of South Australia, Adelaide, South Australia Australia; 2 Faculty of Medicine and Health, University of Adelaide, Adelaide, South Australia Australia

**Keywords:** Flii, Flightless I, Tenocyte, Tendon, Adhesions

## Abstract

**Background:**

Surgical repair of tendons is common, but function is often limited due to the formation of flexor tendon adhesions which reduce the mobility and use of the affected digit and hand. The severity of adhesion formation is dependent on numerous cellular processes many of which involve the actin cytoskeleton. Flightless I (Flii) is a highly conserved cytoskeletal protein, which has previously been identified as a potential target for improved healing of tendon injuries. Using human in vitro cell studies in conjunction with a murine model of partial laceration of the digital flexor tendon, we investigated the effect of modulating Flii levels on tenocyte function and formation of adhesions.

**Methods:**

Human tenocyte proliferation and migration was determined using WST-1 and scratch wound assays following Flii knockdown by siRNA in vitro. Additionally, mice with normal and increased levels of Flii were subjected to a partial laceration of the digital flexor tendon in conjunction with a full tenotomy to immobilise the paw. Resulting adhesions were assessed using histology and immunohistochemistry for collagen I, III, TGF-β1and -β3

**Results:**

Flii knockdown significantly reduced human tenocyte proliferation and migration in vitro. Increasing the expression of Flii significantly reduced digital tendon adhesion formation in vivo which was confirmed through significantly smaller adhesion scores based on collagen fibre orientation, thickness, proximity to other fibres and crimping. Reduced adhesion formation was accompanied with significantly decreased deposition of type I collagen and increased expression of TGF-β1 in vivo.

**Conclusions:**

These findings suggest that increasing the level of Flii in an injured tendon may be beneficial for decreasing tendon adhesion formation.

## Background

Tendon repair is a tightly orchestrated cascade of cellular events that results in the restoration of tendon continuity [1, 2]. The function of the restored tendon is highly dependent on the size and severity of adhesions that form during the healing process as a consequence of the significant inflammatory response that occurs after injury [3]. Formation of adhesions leads to the flexor tendons being bound to each other and surrounding structures hence preventing normal gliding actions [4–6]. For a tendon to heal effectively with its function intact, there must be a re-establishment of strength and gliding functions with minimal adhesion formation [7]. In order for this to occur, a delicate balance must exist between intrinsic and extrinsic repair processes, allowing migration and proliferation of cells to the site of injury to allow effective tendon repair [7–10]. The actin cytoskeleton is important in facilitating the cellular migratory and proliferative processes and proteins that regulate the actin cytoskeleton are integral to the repair process [11–13].

Flightless I (Flii) is an actin remodelling protein, which has been well defined as a negative regulator of cutaneous wound healing [14–16]. Mice with decreased Flii expression (*Flii*^*+/−*^) show improved wound repair and enhanced re-epithelialization, whilst mice overexpressing Flii (*Flii*^*Tg/Tg*^) have impaired repair responses and delayed recovery of the intact skin barrier post injury [14–17]. In contrast to its role in dermal wound repair, Flii has also been shown to positively influence the regeneration of hair follicles [18], and digit regeneration has been observed in *Flii*^*Tg/Tg*^ mice following severe proximal amputation of murine claws [19]. Recent studies have further shown that explanted tendons from Flii overexpressing mice have significantly elevated tenocyte outgrowth compared to *Flii*^*+/−*^ mice [20]. In addition, there is a large body of evidence showing the importance of Flii in cellular adhesion and matrix remodelling [21–23] as well as tissue inflammation during wound healing and in inflammatory skin conditions [24–28], consequently, here, we investigated the effect of altering Flii expression in vivo on tendon adhesion formation. A murine model of tendon adhesion was used in these studies and conducted using a partial laceration of the digital flexor tendon in the third and fourth digit of both hind-paws, in conjunction with a full tenotomy to immobilise the digits of the paw [29]. Although in vitro studies are excellent for fundamental research studies, it is essential to study these concepts in a whole-body environment to establish an understanding of the complex molecular and cellular events involved in tissue repair. Using animals to model human clinical pathologies requires the assumption that the basic pathological processes are similar enough across species to allow comparisons to be made. If these validations are incomplete, it becomes impossible to translate results across species into human clinical solutions. The murine model of tendon adhesion used in these studies is well established [29] and mice limbs and paws are frequently utilised due to the structural and biological similarity to the human hand. In this study, the effect of increased expression of Flii on tendon adhesion formation was investigated using a murine tendon injury model. Histological and immunohistochemical analysis of the tendons and the resulting adhesions was performed. The findings suggest that increasing Flii levels may be beneficial for reducing tendon adhesion formation.

## Methods

### siRNA knockdown of Flii

**Fig. 1:**
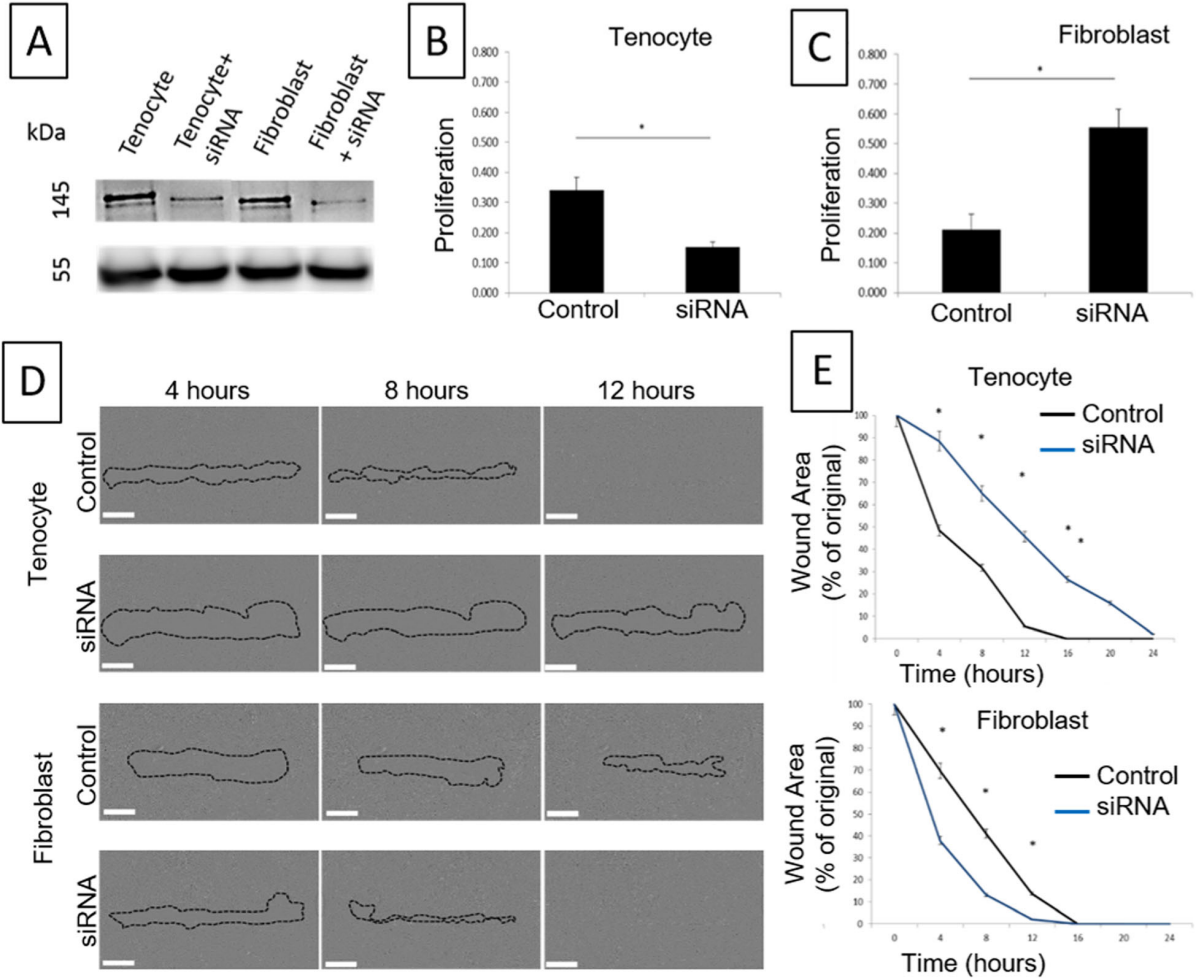
Reduced Flii levels impair human tenocyte cell proliferation and migration. (**a**) Expression of Flii protein in human tenocytes and fibroblasts treated with siRNA or control illustrating reduced Flii levels in response to treatment. Effect of attenuated Flii expression in human tenocytes and fibroblasts using siRNA and subsequent effects on cell proliferation (**b-c**) and migration (**d-e**) assessed using a WST-1 proliferation assay or scratch wound assay respectively. *p ≤ 0.05. Data represented as mean ± SEM. *n* = 6

Flii siRNA transfection was undertaken following optimised experiments described previously [30]. Briefly, human fibroblasts and tenocytes were seeded in triplicate at 30-50% confluency on collagen-coated 24 well plates containing 500 μL DMEM and 20% FCS without antibiotics. Transfection efficiency was optimised using Block-iT fluorescent oligo in the presence of Lipofectamine 2000. Next, cells were seeded on collagen-coated plates for 24 h before Flii and scrambled siRNA were transfected into the cells using Lipofectamine 2000. siRNA was diluted in Opti-MEM I reduced serum media to a final concentration of 60 nM and then incubated 20 min at room temperature with Lipofectamine 2000 to form siRNA:Lipofectamine complex. A total of 500 μl was added to each culture well and incubated with cells for 6 h before replacing transfection media with 10% FCS-supplemented DMEM. Cells were incubated for 48-72 h for gene knockdown assessment, and protein was collected for standard Western blotting experiment demonstrating reduction in Flii expression following previously described protocols [15] (results demonstrated in Fig. 1a). Following optimisation of the protocol, siRNA-treated human tenocytes and fibroblasts were used in proliferation and migration assays described below. No treatment control cells were labelled as control. The oligo nucleotides used in this study are as follows:

Flii siRNA: sense 5′ → GCUGGAACACUUGUCUGUGTT → 3′

(GenePharma, Suzhou, China), antisense 5′ → CACAGACAAGUGUUCCAGCTT → 3′

(GenePharma, Suzhou, China); scrambled siRNA sense 5′ → UUCUCCGAACGUGUCACGUTT → 3′ (GenePharma), antisense 5′ → ACGUGACACGUUCGGAGAATT → 3′

(GenePharma, Suzhou, China); Block-iT (Invitrogen, Carlsbad, USA).

### Proliferation assay

Human tenocytes and fibroblasts were used in a WST-1 metabolic proliferation assay, and transfected with Flii siRNA following established protocols [15]. Cells were cultured until confluent in a 37 °C, 5% CO2 incubator before seeding into 96-well plates at a density of 4 × 10^4^ cells/well. After 24 h, the media was replaced with serum-free DMEM and incubated for 6 h to synchronise the cell cycle, and proliferation was assessed according to manufacturer’s protocols (Roche Applied Science, Munich, Germany). Briefly, 10 μl of WST-1 reagent was added to the cells and left at 37 °C for 30 min. The presence of the formazan product was quantified using a dual absorbance of 450 nm and 600 nm using a plate reader.

### Migration assay

Human tenocytes and fibroblasts were used in a scratch wound assay and transfected with Flii siRNA following established protocols [15]. Cells were plated into 96-well plates and allowed to reach confluence before being wounded using a Woundmaker™ (Essen Bioscience, Michigan, USA) which creates a uniform wound of 700-800 μM in each well of the 96 well ImageLock^TM^ plate. Cells were placed into an Incucyte^TM^ (Essen Bioscience, Michigan, USA) at 37 °C and 5% CO_2_ where images were automatically taken every 3 h for 24 h. The resulting images were analysed using Image Pro Plus 7.1 to determine the effect of Flii siRNA on cell migration.

### Murine surgery

All experiments and maintenance of mice were conducted according to Australian Standards for Animal Care under protocols approved by the Women’s and Children’s Health Network Animal Ethics Committee (WCHN) and carried out in accordance with the Australian code of practice for the care and use of animals for scientific purpose (AE952/9/2016).

### *Flii*^*Tg/Tg*^ mouse generation

All mouse strains were congenic on the Balb/c background and Balb/c littermates were used as wild-type (WT) control animals. Transgenic Flii overexpressing mice (strain name: (Tg1FLII) 2Hdc) were generated as described previously [15] by incorporating a 17.8-Kb fragment of a human cosmid clone that spans the entire FLII locus, with animals homozygous for the transgene in addition to the endogenous Flii allele designated *Flii*^*Tg/Tg*^ [31]. This transgenic strain was backcrossed to Balb/c animals for 10 generations before being intercrossed and homozygous animals were identified by PCR. Following this, the colony was maintained by crossing animals which were identified as homozygous for the transgene. These animals carry two copies of the mouse *Flii* gene and two additional copies of the human *Flii* gene (*Flii*^*Tg/Tg*^) results in elevated levels of Flii protein in various tissues [15, 32]. *Flii*^*Tg/Tg*^ mice are viable and reproduce normally with an average litter size of 7.0 [32].

### Digital tendon adhesion model

Digital tendon adhesion surgery was performed on WT and *Flii*^*Tg/Tg*^ mice following an established model [29]. Humane endpoints were at 3, 7, 14, 21 and 28 days post-surgery (*n* = 6 per group/timepoint). Each mouse received a 50% partial laceration of the digital flexor tendon to the third and fourth digit of both the right and left hind paw (Fig. 1a). Using a Nikon Trinocular dissecting microscope, the digital flexor tendon was exposed through a transverse skin incision and a standardised partial laceration was performed between the A1 and A3 pulley over the proximal phalanx. The skin was closed over the wound with a single 7/0 silk suture. Following the partial laceration of the digital flexor tendons, a further skin incision was made distal to the ankle joint of the left hind limb and a proximal tenotomy performed of the deep and superficial flexor tendons. The incision was closed with two single 7/0 sutures. The tourniquet was removed, and mild pressure applied to restore blood flow in the foot. The mice were then given a single injection of Temgesic (buprenorphine 0.03 mg/kg) for analgesia. Upon recovery, the mice were visually inspected to ensure mobilisation of the right hindlimb and complete immobilisation of the left hindlimb.

### Digit processing

Upon completion of the prescribed endpoint, mice were humanely euthanized and both hind feet were cleaned with 70% ethanol and removed intact, proximal to the ankle, placed into a 6-well plate and covered with 10% buffered formalin overnight. The bone was decalcified in 5% EDTA in 1× PBS for 5 days on a plate shaker. The EDTA was washed out on the fifth day in 3 changes of PBS × 2 min. Feet were placed in 70% ETOH and processed by dehydration in graduated alcohol washes (70% for 2 h, 80% for 1 h, 90% for 1 h, 95% for 1 h and 100% for 3 h). The tissue was then cleared in xylene for 3 h, followed by paraffin wax infiltration under pressure for 4 h at 62 °C. Upon completion of processing, the appropriate toes were dissected from the feet and embedded in paraffin wax [29].

### Histology

**Fig. 2:**
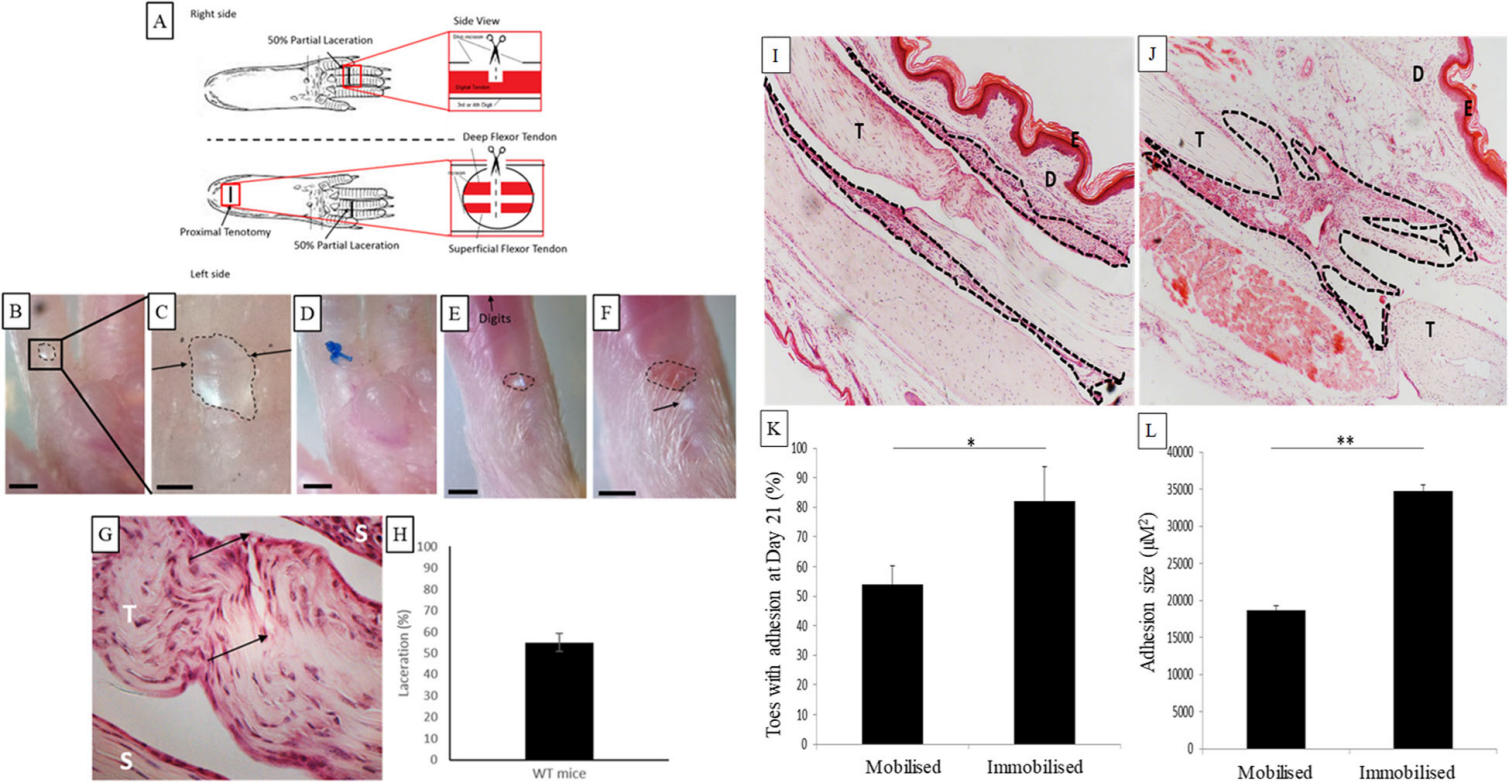
Murine digital tendon adhesion model. (**a**) Schematic of surgical techniques used for murine digital tendon adhesion model. (**b**) Image of 50% laceration surgery in fourth (shown) digit between the A1 and A3 pulleys through a transverse skin incision. Dotted line indicates the exposed tendon. Scale bar = 2 mm. (**c**) A 50% partial laceration (visible between the arrows) is carried out in the exposed tendon. Dotted line indicates the exposed tendon. Scale bar = 500 μM. (**d**) The skin over the laceration is closed with a single suture. Scale bar = 2 mm. (**e**) The deep and superficial flexor tendons are exposed distal to the ankle in the left hind limb by a transverse skin incision. The superficial flexor tendon can be visualised as the white area within the dotted lines. Scale bar = 2 mm. (**f**) A tenotomy is performed, fully dividing the deep and superficial tendons. The ends are buried, the arrow indicates the buried superficial flexor tendon still visible under the skin. Dotted line indicates transverse skin incision. Scale bar = 1 mm. Scale bar = 2 mm. (**g**) H&E image showing 50% of divided tendon fibres (arrows). T indicates the tendon and S the surrounding structures. Magnification × 4, scale bar = 200 μM. (**h**) Graphical representation of laceration validation. *n* = 6. Immobilisation vs mobilisation adhesion formation validation was performed. H&E stained section of tendon in WT mouse 21 days after partial laceration and (**i**) freely mobilised (**j**) immobilised. Dotted lines indicate adhesions. T, tendon; E, epidermis; D, dermis. Magnification × 4. (**k**) Graphical representation of the percentage of WT digits that formed adhesions at D21 in both mobilised and immobilised paws ± SEM. *n* = 6. (**l**) Graphical representation of adhesion size in WT digits at D21 in both mobilised and immobilised paws ± SEM. *n* = 6

Paraffin-embedded samples were sectioned (4 μm) for histological assessment and stained with haematoxylin and eosin (H&E) or Masson’s trichrome staining as described previously [33, 34]. Digits were cut transversely for 400 μm to reach the tendon, following this, 4 μm sections were cut and visualised to ensure consistent sectioning. The epidermis was included to aid orientation, and care was taken to include the damaged tendon area. In some orientations, the tendon may appear to be completely lacerated in the histological sections; however, as demonstrated in Fig. 2, tendons were partially lacerated, and this visual phenomenon may be due to structure of the tendon (i.e. large and small tendon bundles) or orientation of the sample.

### Immunohistochemistry

Immunohistochemical experiments were undertaken on all digits collected from Balb/c WT and Flii^Tg/Tg^ mice at days 0, 3, 7, 14, 21 and 28 post-surgery. Following antigen retrieval, 3% normal goat serum diluted in PBS was used for blocking for 30 min. Primary antibodies were used at 2 μg/ml and included mouse α-Flii (Santa Cruz sc-21716), rabbit α-Collagen I (Rockland 600-401-103), rabbit α-Collagen III (Rockland 600-403-105), mouse α-TGFβ1 (Santa Cruz sc-52893) and rabbit α-TGFβ3 (Santa Cruz sc-83). Species-specific Alexa Fluor 488, 568 or 633-conjugated secondary antibodies (1:400, Invitrogen, Carlsbad, USA) were diluted in PBS and applied for detection. Nuclear counterstain 4,6-diamidino-2-phenyindole (DAPI) was applied last. The slides were mounted in Dako Fluorescent Mounting Medium (DAKO Corporation, Sydney, Australia) and viewed using an Olympus Epifluorescent microscope.

### Image analysis

H&E staining of the tendons were analysed microscopically. Adhesion size was determined by measuring the total size of the adhesion around the tendon using the Image Pro-Plus 7.0 programme (Media Cybernetics Inc, Rockville, USA). Any adhesion formation seen at 4 × magnification was deemed as positive for adhesion formation. Masson’s trichrome stained slides were analysed for collagen formation. Briefly, a macro was created using Image Pro-Plus 7.0 which calculated the number of blue/green (collagen) pixels vs red (muscle/connective tissue) pixels within the tendon/adhesion. As tendon adhesions are made up of 99% collagen type I, this was used as a confirmation of adhesion formation. The adhesions were scored out of a maximum of 10 points for collagen fibre orientation, thickness, proximity to other fibres and crimping. Zero points would indicate an adhesion that had the same collagen structure as a normal tendon. Immunohistochemical samples were analysed by determining fluorescence intensity using the AnalySIS software (Soft-Imaging System GmbH, Munster, Germany). For verification purposes, negative control sections were included in each experiment which excluded staining with the primary antibody or secondary antibody. All negative control sections had negligible immunofluorescence

### Statistical analysis

Data was analysed using the Student’s *t* test to compare between two groups or an ordinary two-way ANOVA with Tukey’s multiple comparison test when comparisons between more than 2 groups were required. A *p* value of < 0.05 was considered significant.

## Results

### Attenuation of Flii using siRNA decreases human tenocyte proliferation and migration

To investigate if human tenocytes and fibroblasts respond equivalently to Flii, siRNA was used to decrease gene expression and the resulting effects on cell proliferation and migration were determined. Flii expression was successfully decreased in both cell types following siRNA treatment (Fig. 1a). Reducing Flii expression in human tenocytes significantly decreased cell proliferation (*p* = 0.0007) (Fig. 1b). The opposite effect was observed in human fibroblasts with significantly increased proliferation observed following Flii knockdown through siRNA transfection (*p* = 0.0001) (Fig. 1c). Reducing Flii in human tenocytes significantly delayed migration, shown using the in vitro scratch wound assay (Fig. 1d-e), but improved fibroblast migration was observed (Fig. 1d-e). The impairment in human tenocyte migration following siRNA treatment (Fig. 1 d-e) was evident at all time points measured (*p* = 0.0004, 0.009, 0.0001, 0.0002 and 0.001 respectively).

### Increasing Flii levels decreases digital flexor tendon adhesions in vivo

Considering that reducing Flii expression showed impaired properties of human tenocytes, we proceeded to investigate the effect of Flii over-expression using a murine model of digital tendon adhesion formation. Mice with normal and increased levels of Flii were subjected to a partial laceration of the digital flexor tendon in the third and fourth digit of both hind paws in conjunction with a full tenotomy to immobilise the paw (Fig. 2a-f). Tendon rupture is extremely uncommon in this model and was not encountered in the mice. To ensure reproducibility of the 50% partial laceration, the divided tendons were stained with H&E and analysed (Fig. 2g). Partial lacerations were performed in both genotypes, and no difference in fibre division was detected between mice. The partial lacerations were found to have an average of 53% ± 4.3% fibre division (Fig. 2h), which was reproducible and robust as a standardised model. Immediately following the partial laceration, the skin was closed over the injury using a single 7/0 silk suture (Fig. 2d). A proximal tenotomy was also performed in the left limb alone, distal to the ankle joint to confirm the superficial and deep tendons could be accessed and divided (Fig. 2e, f). This caused complete immobilisation of the digits of the affected limb but did not affect the mouse’s ability to move around. The effect of the proximal tenotomy on tendon adhesion formation was assessed at day 21 where it was observed that 54% ± 6.2 of the right digits with mobilisation had formed adhesions when compared with 82% ± 11.7 of the left digits with immobilisation (*p* = 0.046) (Fig. 2i-l). Adhesions were significantly larger (*p* = 0.001) at day 21 in mice with immobilised digits 34697.83μM^2^ ± 863.3 when compared with mobilised digits 18654.44 μM2 ± 570.3 (Fig. 2l) and these were used for analysis in the rest of the study.

### *Flii*^*Tg/Tg*^ mice have impaired cutaneous healing but significantly smaller digital tendon adhesions than WT mice

**Fig. 3:**
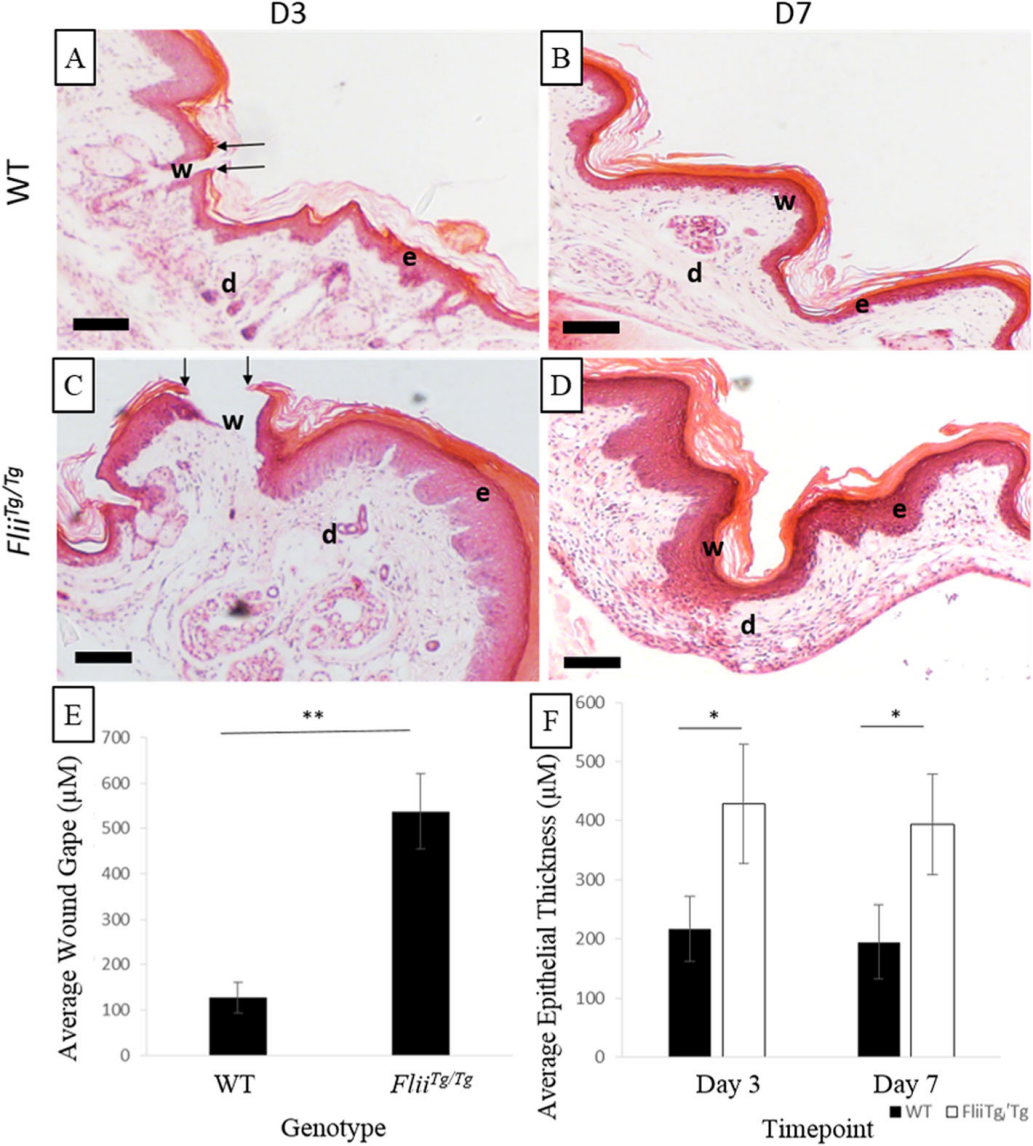
*Flii*^*Tg/Tg*^ mice have slower skin re-epithelialisation and increased epidermal thickness. (**a-d**) Representative images of H&E stained digits, showing the area of skin injury carried out to allow access to the tendon in WT and *Flii*^*Tg/Tg*^ mice at days 3 and 7. Images illustrate the significantly delayed re-epithelialisation and increased epidermal thickness in *Flii*^*Tg/Tg*^ mice compared with WT mice. W, wound; D, dermis; E, epidermis. Arrows indicate edge of wound. Magnification × 10. Scale bar = 200 μM. *n* = 6. (**e**) Graph showing average wound gape at day 3 in WT and Flii^Tg/Tg^ mice. (**f**) Graph showing average epidermal thickness of WT and Flii^Tg/Tg^ mice at days 3 and 7. *n* = 6, mean ± SEM. *p ≤ 0.05, **p ≤ 0.01

**Fig. 4:**
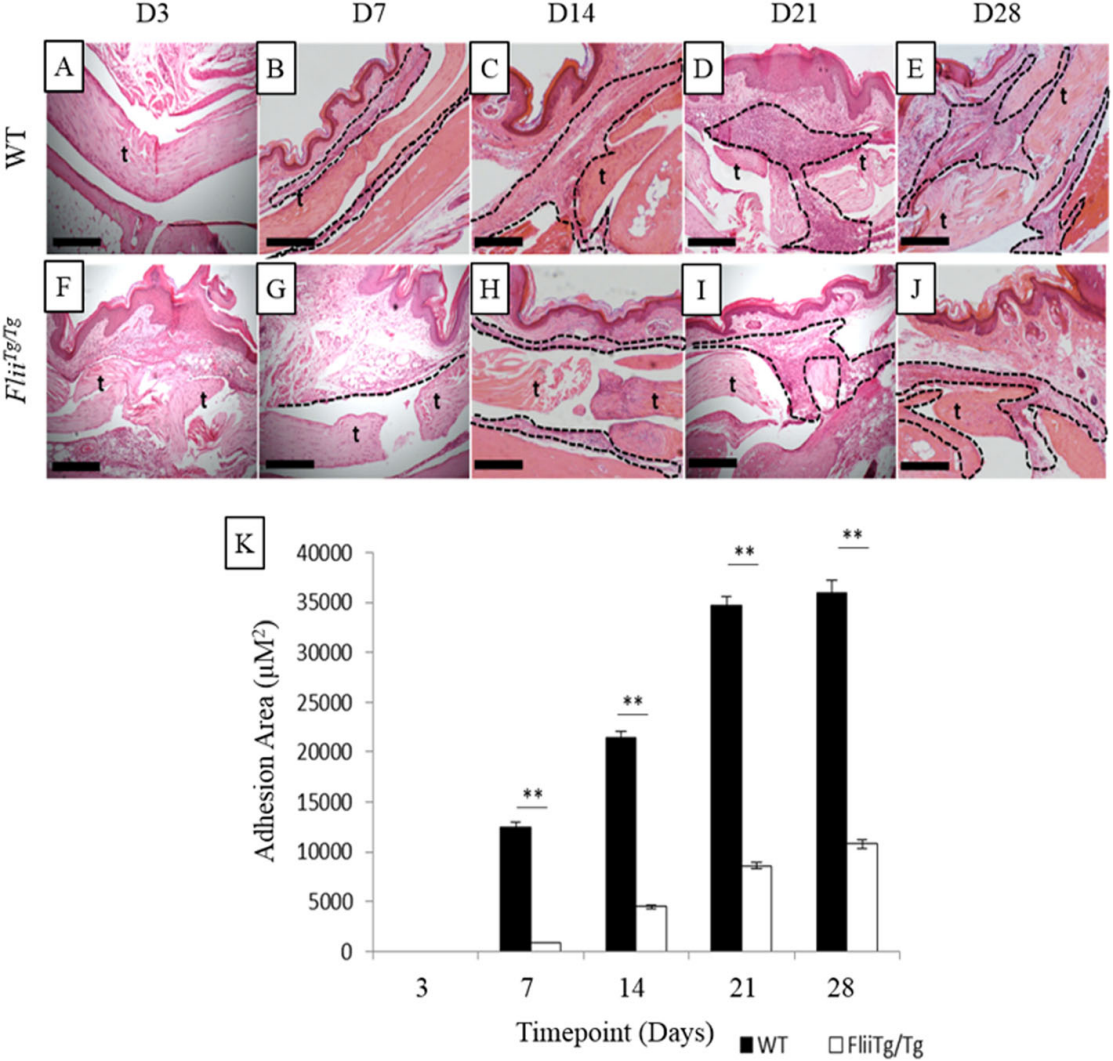
*Flii*^*Tg/Tg*^ mice form smaller adhesions following a 50% partial laceration tendon injury compared with WT counterparts. Representative images of H&E stained digits showing the area of tendon injury and the surrounding adhesions and structures in WT (**a-e**) and *Flii*^*Tg/Tg*^ (**f-j**) mice. (**k**) Graphical representation of adhesion size following a 50% partial laceration, on days 3, 7, 14, 21 and 28 following injury. *n* = 6. Data represented as mean ± SEM. **p ≤ 0.01. t, tendon; dotted line, adhesion area. Magnification × 4. Scale bar = 200 μM

The effect of Flii expression on digital tendon adhesion formation in immobilised paws was investigated using H&E stained sections of mice hind-paw digital tendons injured with a 50% laceration and harvested at days 3, 7, 14, 21 or 28 using 12-week-old female WT or *Flii*^*Tg/Tg*^. The injury to the skin that occurred to access the tendon and sheath was also assessed and revealed that *Flii*^*Tg/Tg*^ mice had significantly slower re-epithelialisation than WT mice (*p* = 0.011) (Fig. 3a-f) and a significantly thickened epidermis on days 3 and 7 (*p* = 0.045 and *p* = 0.049 respectively) (Fig. 3f). Digital tendon adhesions in WT and *Flii*^*Tg/Tg*^ mice were measured at days 3, 7, 14, 21 and 28 post 50% laceration injury. Adhesions were recognised as a fibrotic mass between the tendon and surrounding structures with architecture visually different to the dermal and tendon structures (Fig. 4a-j). *Flii*^*Tg/Tg*^ mice formed significantly smaller adhesions than WT mice on days 7, 14, 21 and 28 post-injury (*p* = 0.0003, 0.0006, 0.0007 and 0.0003 respectively) (Fig. 4k).

### Flii is upregulated in *Flii*^*Tg/Tg*^ tendon adhesions

**Fig. 5:**
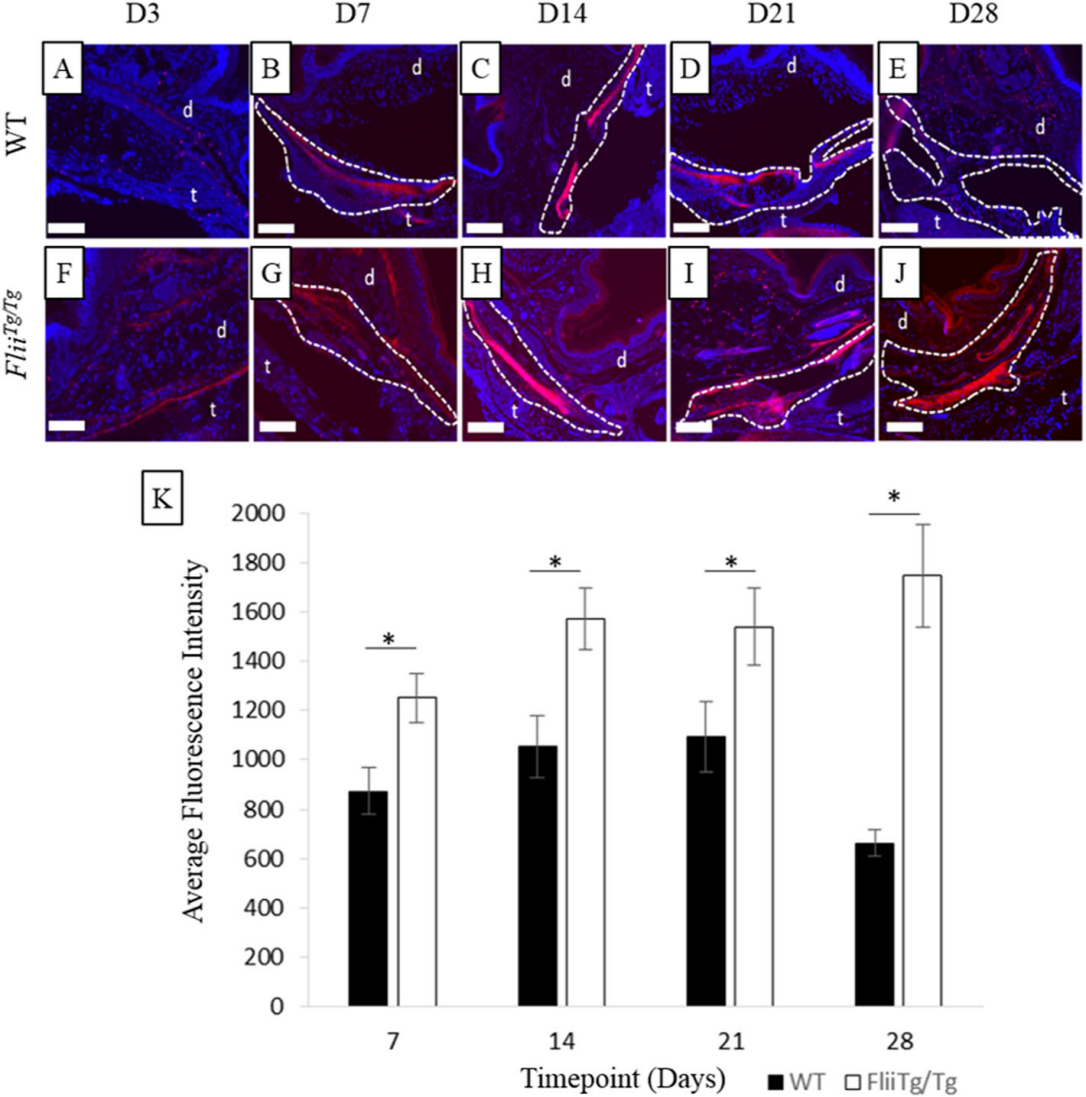
Adhesions in *Flii*^*Tg/Tg*^ mice have upregulated Flii levels. Representative images immunostained for Flii expression in WT (**a-e**) and Flii^Tg/Tg^ (**f-j**) mice from days 3, 7, 14, 21 and 28 post-injury. Flii expression is detected as red fluorescence and DAPI staining detected as blue fluorescence. t, tendon; d, dermis; dotted line, adhesion area. Magnification × 10. Scale bar = 200 μM and refers to all images. (**k**) Graphical representation of Flii expression in the tendon adhesions of WT and *Flii*^*Tg/Tg*^ mice at 7, 14, 21 and 28 days post 50% partial laceration injury. No adhesions were detected at day 3 post injury. *n* = 6. Data represented as mean ± SEM. *p ≤ 0.05

The expression of Flii was assessed using immunohistochemistry and found to be significantly upregulated in the adhesions of *Flii*^*Tg/Tg*^ mice at days 7, 14, 21 and 28 post 50% laceration injury compared with WT mice (*p* = 0.047, 0.038, 0.038, 0.024 respectively) (Fig. 5a-k). Flii expression peaked in the tendon adhesions of WT mice at day 21 and in the adhesions of *Flii*^*Tg/Tg*^ mice at day 28 (Fig. 5a-k).

### *Flii*^*Tg/Tg*^ mice have slower, more organised collagen deposition and reduced adhesion score

**Fig. 6:**
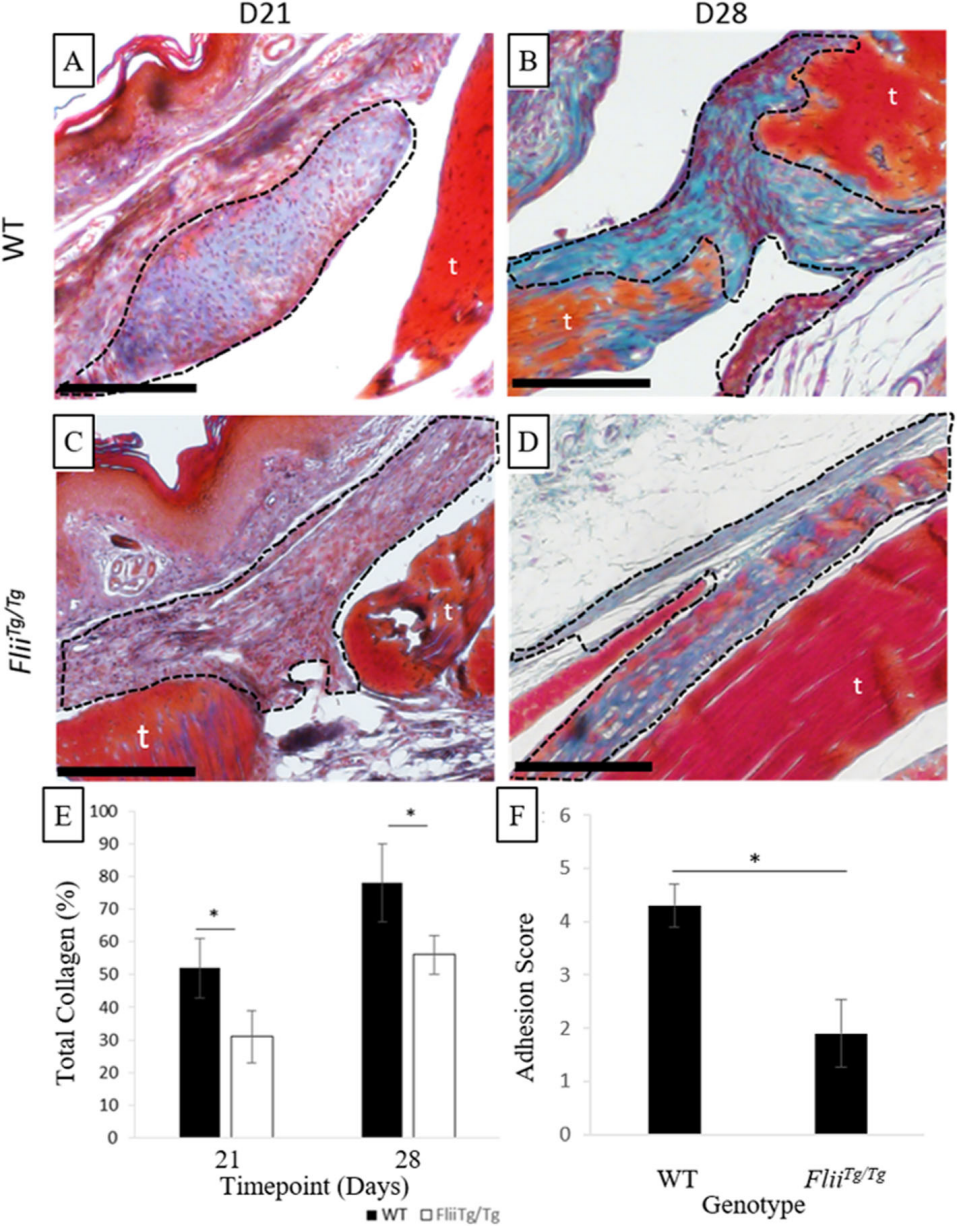
*Flii*^*Tg/Tg*^ mice have slower, more organised collagen deposition within the adhesion and significantly decreased adhesion score. (**a-d**) Representative images of tendon adhesions in WT (**a-b**) and *Flii*^*Tg/Tg*^ (**c-d**) mice at days 21 and 28 post-injury, stained with Masson’s trichrome. Dotted line represents adhesion area; t, tendon. Magnification × 20, scale bar = 200 μM. (**e**) Graph showing significantly decreased collagen deposition in the healing tendons of *Flii*^*Tg/Tg*^ mice compared with WT at day 21 and 28. (**f**) Graph showing decreased adhesion score indicative of collagen fibres appearing more similar to those of unwounded tendons. *n* = 6. Data represented as mean ± SEM. *p ≤ 0.05

Masson’s trichrome staining was used to differentiate between connective tissues such as collagen and cytoplasm. A digital macro was used to quantify red vs. green staining at days 21 and 28 post-injury. It was determined that *Flii*^*Tg/Tg*^ mice had significantly lower collagen deposition in the healing tendon than WT mice (*p* = 0.048 and 0.044 respectively) (Fig. 6a-e). Adhesions in *Flii*^*Tg/Tg*^ mice had a reduced adhesion score with significantly more organised collagen fibres than those of WT mice (*p* = 0.05) (Fig. 6f). *Flii*^*Tg/Tg*^ adhesions were more consistent with the organisation seen in the uninjured tendon, with fibres following a crimped wavy path and appearing similar in structure to normal tendons.

### *Flii*^*Tg/Tg*^ mice adhesions commence remodelling process slower than WT mice adhesions

**Fig. 7:**
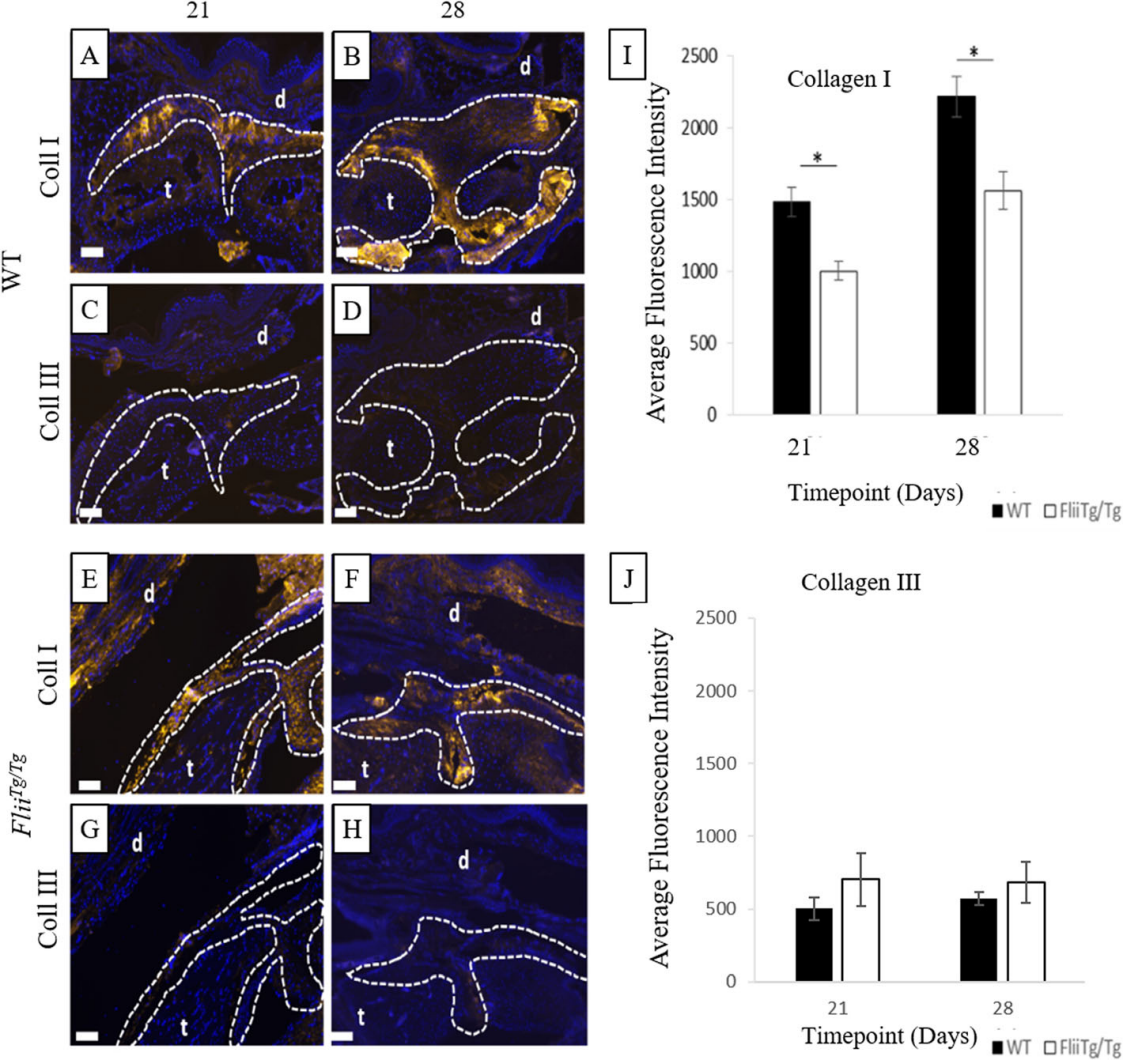
WT mice have significantly increased collagen type I expression in the tendon adhesions. Representative images of collagen type I expression (gold) in the tendon adhesions of WT (**a-b**) and *Flii*^*Tg/Tg*^ (**e-f**) mice and collagen type III expression (gold) in the tendon adhesions of WT (**c-d**) *Flii*^*Tg/Tg*^ (**g-h**) at 21 and 28 days post 50% partial laceration injury. (**i**) WT mice have significantly upregulated collagen type I levels in the adhesions compared with *Flii*^*Tg/Tg*^ mice. (**j**) No significant difference was noted in collagen type III levels in WT and *Flii*^*Tg/Tg*^ mice, although detectable levels of collagen type III were found in the *Flii*^*Tg/Tg*^ mice. DAPI is represented as blue fluorescence; t, tendon; d, dermis. Dotted line represents tendon adhesion area. Magnification × 10. Scale bar = 200 μM and refers to all images. Data represented as mean ± SEM. *p ≤ 0.05. *n* = 6

Immunohistochemical staining allowed the investigation of levels of collagen type I and III (Coll I and III respectively) in the established adhesions at days 21 and 28 (Fig. 7). Whilst unwounded tendons and established adhesions are composed primarily of Coll I, Coll III is secreted by tenocytes in the early stages of the healing process [35]. Comparing the levels of these two collagens allowed the investigation of the rate of the healing process and the severity of adhesion formation. WT mice had significantly increased Coll I in the adhesions at days 21 and 28 compared with the *Flii*^*Tg/Tg*^ mice (*p* = 0.044 and 0.032 respectively) (Fig. 7a-i). *Flii*^*Tg/Tg*^ mice still had low levels of Coll III present in the adhesion at days 21 and 28 whereas WT mice had no Coll III detectable (Fig. 7j).

### *Flii*^*Tg/Tg*^ mice adhesions express higher levels of TGFβ1 and lower levels of TGFβ3 than WT mice

**Fig. 8:**
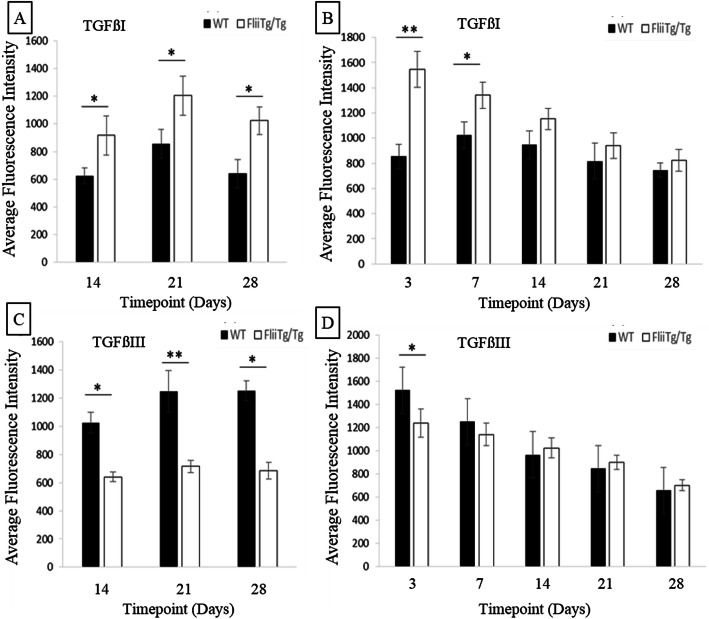
*Flii*^*Tg/Tg*^ mice adhesions express higher levels of TGFβ1 and lower levels of TGFβ3 than WT mice. (**a**) TGFβ1 expression levels in the tendon adhesions and (**b**) surrounding dermis show significantly elevated expression in *Flii*^*Tg/Tg*^ mice compared with WT mice. (**c**) TGFβ3 expression levels in the tendon adhesions and (**d**) surrounding dermis show significantly elevated expression in WT mice compared with *Flii*^*Tg/Tg*^ mice. Data represented as mean ± SEM. *p ≤ 0.05, **p ≤ 0.01. *n* = 6

TGFβs are a family of important growth factors known to be active in all the phases of tendon healing. TGFβ isoforms are involved in a variety of healing processes including collagen production and intrinsic/extrinsic cell migration. Flii has been shown to regulate TGFβ expression [36], and increased Flii levels have been shown to upregulate the pro-fibrotic TGFβ1 isoform and downregulate the anti-scarring TGFβ3 isoform in cutaneous wounds [25, 36]. Here, the expression of TGFβ1 and TGFβ3 in the adhesions and surrounding dermis in WT and *Flii*^*Tg/Tg*^ mice was investigated using immunohistochemistry. TGFβ1 expression in tendon adhesions was significantly higher in *Flii*^*Tg/Tg*^ mice than WT mice, at days 14, 21 and 28 (*p* = 0.048, 0.044 and 0.039 respectively) with expression in both genotypes peaking at day 21 post injury (Supplementary Figure 1 and Fig. 8a). TGFβ1 expression in the surrounding dermis was also significantly elevated in *Flii*^*Tg/Tg*^ mice at days 3 and 7 (*p* = 0.01 and 0.046 respectively) when compared with WT mice, with expression peaking at days 3 and 7 post injury respectively (Fig. 8b). In contrast, TGFβ3 expression was significantly elevated in both the adhesion at days 14, 21 and 28 (*p* = 0.033, 0.009 and 0.018 respectively) (Fig. 8c) and the dermis in WT mice at day 3 (*p* = 0.044) when compared with *Flii*^*Tg/Tg*^ mice (Fig. 8d). Both genotypes had peak expression of TGFβ3 in the adhesion at day 21 and in the surrounding dermis at day 3 post injury (Fig. 8c-d).

## Discussion

Tendon injuries require a complex re-organisation of the actin cytoskeleton in order to facilitate essential processes for repair including migration and proliferation whilst minimising tendon adhesions which hinder repair and functionality. Tenocytes make up the majority of the cellular content in tendons and are integral in coordinating the healing process following injury including release of signalling molecules to recruit a variety of growth factors, cytokines and inflammatory cells to the injury site in order to facilitate successful healing [37]. We have previously shown the effect of differential *Flii* gene expression on murine tenocyte function in vitro [20]. In this study, we investigated the effect of modulating Flii levels on human tenocyte function and formation of tendon adhesions using a murine model of a partial laceration of the digital flexor tendon.

Flii belongs to a family of actin remodelling proteins critical in modulating cellular responses during development, tissue healing and regeneration [16, 17, 21, 38]. Previous studies have shown that Flii in expressed in murine tenocytes and its expression is upregulated in response to wounding using in-vitro models [20]. In order to ascertain the role of Flii in tendon adhesion formation, we first investigated the effects of reducing Flii expression on human tenocyte and fibroblast function. In agreement with previous studies [15], we found that reducing Flii using siRNA resulted in improved human fibroblast cell proliferation and migration properties in vitro. This however contrasted with human tenocytes where reduction of Flii leads to negative cellular responses including reduced tenocyte proliferation and migration.

Digital tendon adhesions form as the result of a large inflammatory response following injury and result in the tendon being bound to surrounding structures, preventing normal gliding function and significantly reducing the mobility and function of the affected digit and the hand [39–41]. To determine the role of Flii in adhesion formation, a murine model of digital tendon adhesion formation was used with mice with either normal or increased expression of Flii [32]. Overexpression of Flii resulted in significantly smaller tendon adhesions when compared with WT mice, over a period of 28 days suggesting that Flii is important in regulating tendon adhesion formation. The model involves penetrating the tendon sheath, allowing the influx of extrinsic cells from the surrounding tissue which results in the formation of an adhesion. With *Flii*^*Tg/Tg*^ mice showing significantly smaller tendon adhesion size, this suggests that Flii may affect adhesion formation through regulation of extrinsic cell migration to the wound site. Interestingly, an upregulation of Flii expression appears to be positively correlated with decreased adhesion formation which is in contrast to previous research in cutaneous repair which has indicated that Flii negatively regulates cutaneous healing [14, 17, 32, 42]. This may be due to a tissue-specific effect, as previous research has shown that Flii can positively regulate healing in mammals in areas, which have retained a regenerative phenotype including the digit tip [19]. In order to access the tendon, an incision was made through the epidermal and dermal layers of the digit. The cutaneous wound notably had impaired healing in the *Flii*^*Tg/Tg*^ mice compared with WT mice, with slower re-epithelialisation and a significantly thickened epidermis. This shows that the positive effect of Flii seen on adhesion formation was not due to a positional effect, as the skin in the same area retained its negative healing outcomes with increased Flii levels and suggests a cell-specific response to Flii results in the positive response of smaller adhesions in *Flii*^*Tg/Tg*^ mouse tendons.

Immunohistochemical staining of Flii showed a significant increase in Flii expression in the tendon adhesions and surrounding dermis of *Flii*^*Tg/Tg*^ mice, suggesting that any improvements seen in the formation of adhesions were likely due to increased Flii levels. Negligible staining for Flii was observed in intact tendons likely due to their being mainly composed of collagen type I protein. Flii may be expressed in tenocytes within the tendon, but these cells are found sporadically throughout the tendon and would be hard to detect in significant numbers in in vivo cross sections.

Collagen type I is the main component of tendon adhesions and cutaneous scarring [43, 44]. *Flii*^*Tg/Tg*^ mice have significantly more collagen type I in their scars than mice with lower levels of Flii [15]. Established research has shown that although collagen type I is essential for healing and scar strength, the speed of collagen production and the quality of its organisation and remodelling is also vital in determining the physiological outcome of the resulting scar, with slower more organised collagen type I production resulting in a better healing outcome [15]. *Flii*^*Tg/Tg*^ mice had less collagen type I than WT mice in the tendon adhesions. Moreover, these collagen fibres in *Flii*^*Tg/Tg*^ mice appeared closer to that of unwounded tendons. This suggests that during tendon repair, increased Flii levels may cause a more controlled cellular response, leading to slower, more organised collagen type I deposition. The mechanism by which Flii regulates collagen type I deposition is still unknown, but it has been suggested that it may modulate transcription of genes including TGFβ1 in wound healing [15].

Flii has been shown to play a critical role in TGFβ1/SMAD-mediated transcription of collagen which is important for fibrosis and wound repair [25]. Through its role in interacting with the SWI/SNF complex, Flii recruits the TGFβ-responsive element (TRE) in a TGFβ-dependent manner, facilitating TGFβ-induced chromatin accessibility to target genes including the *COL1A2* gene promoter [45]. Flii is also critical for the recruitment of Brahma-related gene 1 (BRG1), a core ATPase associated with SMAD2 and SMAD3, to the *COL1A2* promoter region leading to collagen production [45]. In response to wound healing, Flii translocates from the cytoplasm to the nucleus where it forms a transcription complex with activating proteins-1 (AP-1) c-fos and c-jun which bind to the TGF-β promoter to regulate its expression [25]. Flii may therefore function as a nuclear receptor co-activator through its direct interaction with c-fos and c-jun and/or other co-activator complexes may modulate *TGF-β* gene expression. Flii has further been shown to regulate TGF-β1/SMAD signalling indirectly by suppressing the function of Akt in the nucleus of wounded fibroblasts which is involved in the post-translational phosphorylation of SMADs [36]. TGFβ1 is upregulated shortly after tendon injury, specifically in the tendon sheath and the epitenon [46, 47]. TGFβ1 often leads to increased production of collagen type I subsequently resulting in excessive, disordered collagen type I production, leading to adhesions and fibrosis [48]. Flii modulates TGFβ expression, with decreased levels of TGFβ1 and increased levels of TGFβ3 detected in cutaneous healing studies in Flii^+/−^ mice [36]. Although *Flii*^*Tg/Tg*^ mice adhesions had significantly higher TGFβ1 levels early in the healing process, these peaked at day 3 and dropped off significantly by day 14. In comparison, WT mice adhesions do not see a peak in TGFβ1 levels until day 7, and these levels remain relatively high across the course of healing. This delayed expression of TGFβ1 may help to explain why the formation of adhesions occur earlier with significantly higher collagen type I levels in WT vs *Flii*^*Tg/Tg*^ mice adhesions.

## Conclusions

In summary, this study has shown that overexpression of Flii leads to smaller tendon adhesions with collagen organisation more closely resembling unwounded tendons. If translated into the human situation this could potentially lead to improved mobility and function of an injured digit or hand. Whilst the mechanism for tendon adhesion formation remains to be elucidated, these studies support further investigations aimed at determining if Flii could be a potential target for a therapeutic intervention for decreasing human tendon adhesion formation.

## Supplementary information

**Additional file 1: Figure S1**. TGFβ1 and TGFβ3 expression in WT mice at days 3, 7, 14, 21 and 28 post injury. TGFβ1 and TGFβ3 expression in WT and Flii^Tg/Tg^ mice at days 3, 7, 14, 21 and 28 post injury. Representative images of TGBβ1 expression (A-E), and TGFβ3 expression (F-J) in WT mice from days 3-28 post partial laceration injury. (K-O) are composite images of the two stains. Representative images of TGBβ1 expression (A1-E1), and TGFβ3 expression (F1-J1) in Flii^Tg/Tg^ mice from days 3-28 post partial laceration injury. (K1-O1) are composite images of the two stains. TGFβ1 is represented by red staining, TGFβ3 represented by gold staining and DAPI by blue staining. t = tendon. d = dermis. Dotted line represents tendon adhesion area. Magnification x 10. Scale bar = 200 = μM.

## Data Availability

The datasets used and/or analysed during the current study are available from the corresponding author on reasonable request.

## Abbreviations

AP-1: Activating protein-1
Col: Collagen
DAPI: 4’,6-diamindino-2-phenylindole
EDTA: Ethylediaminetetraacedic acid
ETOH: Ethanol
Flii: Flightless
H&E: Haematoxylin and eosin
PBS: Phosphate buffered saline
TGF-β: Transforming growth factor beta
WT: Wild type
