# Supplementary figures and images for: Increasing the level of cytoskeletal protein Flightless I reduces adhesion formation in a murine digital flexor tendon model
Source: J Orthop Surg Res. 2020 Aug 27;15:362. doi: 10.1186/s13018-020-01889-y (PMC7450967; doi:10.1186/s13018-020-01889-y)

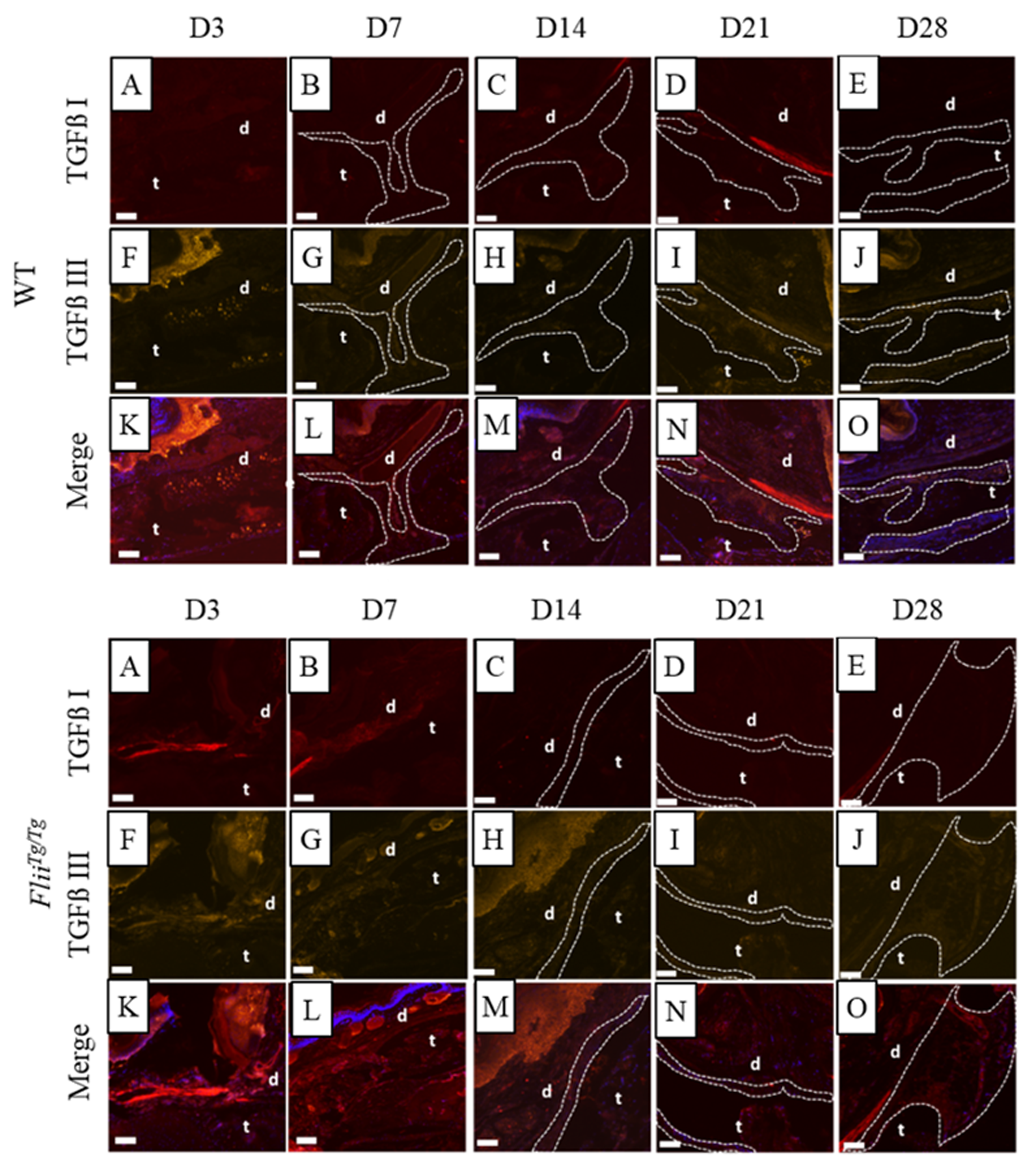

Supplement: Supplementary file 1 — Additional file 1: Figure S1. TGFβ1 and TGFβ3 expression in WT mice at days 3, 7, 14, 21 and 28 post injury. TGFβ1 and TGFβ3 expression in WT and FliiTg/Tg mice at days 3, 7, 14, 21 and 28 post injury. Representative images of TGBβ1 expression (A-E), and TGFβ3 expression (F-J) in WT mice from days 3-28 post partial laceration injury. (K-O) are composite images of the two stains. Representative images of TGBβ1 expression (A1-E1), and TGFβ3 expression (F1-J1) in FliiTg/Tg mice from days 3-28 post partial laceration injury. (K1-O1) are composite images of the two stains. TGFβ1 is represented by red staining, TGFβ3 represented by gold staining and DAPI by blue staining. t = tendon. d = dermis. Dotted line represents tendon adhesion area. Magnification x 10. Scale bar = 200 = μM. [file 13018_2020_1889_MOESM1_ESM.tif]
